# Molecular Evidence for Intra- and Inter-Farm Spread of Porcine *mcr*-*1*-Carrying *Escherichia coli* in Taiwan

**DOI:** 10.3389/fmicb.2020.01967

**Published:** 2020-08-20

**Authors:** Shun-Chung Hsueh, Chih-Cheng Lai, Yu-Tsung Huang, Chun-Hsing Liao, Ming-Tang Chiou, Chao-Nan Lin, Po-Ren Hsueh

**Affiliations:** ^1^Department of Medicine, College of Medicine, Taipei Medical University, Taipei, Taiwan; ^2^Department of Internal Medicine, Kaohsiung Veterans General Hospital, Kaohsiung City, Taiwan; ^3^Division of Infectious Diseases, Far Eastern Memorial Hospital, New Taipei City, Taiwan; ^4^Department of Laboratory Medicine, National Taiwan University Hospital, National Taiwan University College of Medicine, Taipei, Taiwan; ^5^Department of Medicine, National Yang-Ming University, Taipei, Taiwan; ^6^Department of Veterinary Medicine, College of Veterinary Medicine, National Pingtung University of Science and Technology, Pingtung, Taiwan; ^7^Animal Disease Diagnostic Center, College of Veterinary Medicine, National Pingtung University of Science and Technology, Pingtung, Taiwan; ^8^Department of Internal Medicine, National Taiwan University Hospital, National Taiwan University College of Medicine, Taipei, Taiwan

**Keywords:** *Escherichia coli*, sick pigs, colistin resistance, *mcr-1*, genotypes

## Abstract

From January 2013 to December 2018, 90 *Escherichia coli* isolates were collected from 90 sick pigs on 58 farms in seven cities in Taiwan. The minimum inhibitory concentrations (MICs) of the isolates to 14 antimicrobial agents were determined on the VITEK 2 system (bioMérieux, Marcy-l’Etoile, France), and the resistance to colistin was assessed via the reference broth microdilution (BMD) method. The *mobilized colistin resistance* genes (*mcr*) were determined for the colistin-resistant isolates, which displayed BMD MICs ≥ 4 mg/L. Genotypes of the *mcr*-positive *E*. *coli* isolates were determined by multilocus sequence typing, arbitrarily primed polymerase chain reaction (PCR), and pulsed-field gel electrophoresis. All of the isolates were tested for susceptibility to carbapenems. Fifty isolates (55.6%) were resistant to colistin, 39 of which (78%) were positive for the *mcr*-*1* gene. *E*. *coli* isolates harboring *mcr-1* were most frequent in 2017 (15/18, 83.3%), followed by 2018 (13/23, 56.5%), 2015 (7/21, 33.3%), and 2016 (3/24, 12.5%). A total of 18 sequence types (STs) were identified among the 39 porcine *mcr*-*1*-carrying *E*. *coli* isolates; 13 were ST2521 (33.3%) isolated in 2017 and 2018. Five genotypes (clones) were identified, and the same genotypes were in sick pigs on the same farm and different farms. This suggests intra- and inter-farm spread of porcine *mcr*-*1*-carrying *E*. *coli*. The results presented here indicate high colistin resistance and wide *mcr*-*1 E*. *coli* prevalence among the sick pigs sampled in 2015–2018 in different regions of Taiwan.

## Introduction

The plasmid-borne *mobilized colistin resistance-1* gene (*mcr-1*) confers resistance to polymyxin E (colistin) in *Escherichia coli* (Liu et al., 2016) and has been reported on nearly every continent (Nordmann et al., 2016; Meinersmann et al., 2017; Lai et al., 2018; Rebelo et al., 2018). *Enterobacteriaceae* isolates positive for *mcr-1* have been recovered from humans and animals, including chickens, turkeys, swine, and pets (Brennan et al., 2016; Kempf et al., 2016; Kuo et al., 2016; Khan et al., 2017; Pulss et al., 2017; Lai et al., 2018). The gene is readily transferable, which limits the treatment options, especially if it resides in multidrug-resistant organisms (Lai et al., 2017; Pulss et al., 2017; Lin et al., 2018; Palmeira et al., 2018). Previous studies have shown that food animals are common sources of human infections due to colistin-resistance in these organisms (Kuo et al., 2016; Chiou et al., 2017). Moreover, the horizontal transmission of a colistin-resistant *E*. *coli* from a domesticated pig to a boy may have occurred (the boy fed the pig without protective equipment) (Olaitan et al., 2015).

The use of colistin in animal husbandry in Taiwan has been restricted since July 2007, but *mcr-1*-harboring *E*. *coli* isolates are still a concern. Liu et al. (2018) documented high rates of *mcr-1*-harboring *E*. *coli* (mainly enterohemolytic *E*. *coli*) in sick pigs from southern Taiwan. A high rate (46.2%) of colistin resistance among zoonotic *Salmonella* spp. has also been reported (Chiou et al., 2017). The *mcr-1* gene has been identified in humans and retail meats (Kuo et al., 2016; Lai et al., 2018). Kuo et al. (2016) described 18 colistin-resistant *E*. *coli* isolates positive for *mcr-1* in ground beef, chicken, and pork. The prevalence of *mcr-1* in meat-associated *E*. *coli* isolates has increased in Taiwan, with rates of 1.1, 6.6, and 8.7% in 2012, 2013, and 2015, respectively (Kuo et al., 2016).

To our knowledge, no longitudinal studies have examined the prevalence of colistin-resistant *E*. *coli* in sick pigs. This study investigated the annual prevalence of colistin resistance and *mcr*-harboring *E*. *coli* in sick pigs from different farms in Taiwan.

## Materials and Methods

### Bacterial Isolates From Sick Pigs

A total of 90 *E*. *coli* isolates were obtained from 90 sick pigs from 58 farms in different geographical regions of Taiwan between January 2013 and December 2018. A total of 88 isolates were collected from seven counties in southern (*n* = 4), northern (*n* = 1), middle (*n* = 1), and eastern Taiwan (two isolates were from unknown sources; Table 1). Most of the isolates were collected between 2015 and 2018 (*n* = 84, 93.3%), from southern Taiwan (*n* = 82, 91.1%). The age of the sick pigs was known for 79 isolates, 78 (98.7%) were <10 weeks old. Isolates from rectal swabs (*n* = 32) and mesenteric lymph nodes (*n* = 32) comprised 71.2% of the samples. The isolates were sent to the Animal Disease Diagnostic Center, College of Veterinary Medicine, National Pingtung University of Science and Technology, Pingtung, Taiwan, for isolation and identification of the enterohemolytic *E*. *coli*.

**TABLE 1:** Characteristics of the 90 *E*. *coli* isolates from 90 sick pigs sampled in 2013 to 2018 in Taiwan.

Characteristics	No. (%)
**Year of isolation**	
2013	2 (2.2)
2014	2 (2.2)
2015	21 (23.3)
2016	24 (26.7)
2017	18 (20.0)
2018	23 (25.6)
**Farm location**	
Pingtung (southern Taiwan)	51 (56.7)
Yunlin (southern Taiwan)	15 (16.7)
Tainan (southern Taiwan)	9 (10.0)
Kaohsiung (southern Taiwan)	7 (7.8)
Hsinchu (northern Taiwan)	4 (4.4)
Changhua (middle Taiwan)	1 (1.1)
Hualien (eastern Taiwan)	1 (1.1)
Unknown	2 (2.2)
**Age of the sick pigs (weeks)**	
<1	21 (23.3)
1–5	22 (24.4)
6–10	35 (38.9)
11–15	1 (1.1)
Unknown	11 (12.2)
**Isolate source**	
Rectal swab	32 (35.6)
Mesenteric lymph nodes	32 (35.6)
Spleen	6 (6.7)
Small intestine	6 (6.7)
Colon	4 (4.4)
Ileum	2 (2.2)
Duodenum	2 (2.2)
Others^*a*^	6 (6.7)
***E*. *coli* isolates**	
Enterohemolytic	45 (50.0)
2013 (*n* = 2)	0 (0)
2014 (*n* = 2)	0 (0)
2015 (*n* = 21)	10 (47.6)
2016 (*n* = 24)	9 (37.5)
2017 (*n* = 18)	12 (66.7)
2018 (*n* = 23)	14 (60.9)
Non-enterohemolytic	45 (50.0)
Colistin-resistant^*b*^	50 (55.6)
*mcr*-*1* positive	39 (43.3)

*^*a*^Includes one each from the pericardial effusion, testis, meninges, liver, mesentery, and stool. ^*b*^Colistin resistance was determined via the reference broth microdilution method and interpreted from the guidelines of the Clinical and Laboratory Standards Institute (Clinical and Laboratory Standards Institute [CLSI], 2020).*

### Identification of the Enterohemolytic *E*. *coli*

*Escherichia coli* isolates were identified as enterohemolytic strains by the presence of β-hemolysis on Trypticase soy agar supplemented with 5% sheep blood and by the presence of at least one virulent gene (STa, STb, LT, F18, or *aidA*) (Chapman et al., 2006; Lai et al., 2017, 2018).

### Antimicrobial Susceptibility Testing

The minimum inhibitory concentrations (MICs) of 14 antimicrobial agents, including colistin, were determined on the VITEK 2 system (bioMérieux, Marcy-l’Etoile, France) (Table 2) and were interpreted according to guidelines of the Clinical and Laboratory Standards Institute (CLSI). The MICs of colistin were determined via the reference broth microdilution (BMD) method (Clinical and Laboratory Standards Institute [CLSI], 2020). Susceptibility to colistin was defined as intermediate (MICs of ≤2 mg/L) or resistant (MICs ≥ 4 mg/L) as in the MIC interpretive criteria from CLSI.

**TABLE 2:** Susceptibilities of the 90 *E*. *coli* isolates as determined by the VITEK 2 susceptibility method.

Agent	MIC (mg/L)			No. (%) of isolates with the indicated susceptibility category		
	Range	50%	90%	S (%)	I (%)	R (%)
Cefazolin	≤4 to ≥64	≥64	≥64	30 (33.3)	–	60 (66.7)
Ceftazidime	≤0.12 to ≥64	4	16	51 (56.7)	3 (3.3)	36 (40.0)
Cefepime	≤0.12 to ≥64	≤1	2	85 (94.4)	2 (2.2)	3 (3.3)
Piperacillin-tazobactam	0.5 to ≥128	≤4	64	74 (82.2)	15 (16.7)	1 (1.1)
Ertapenem	≤0.06 to 0.5	≤0.5	≤0.5	90 (100.0)	0 (0.0)	0 (0.0)
Imipenem	0.12 to 0.5	≤0.25	0.25	89 (100.0)	0 (0.0)	0 (0.0)
Meropenem	≤0.06 to 0.25	≤0.25	≤0.25	90 (100.0)	0 (0.0)	0 (0.0)
Ciprofloxacin	≤0.06 to ≥64	1	≥4	49 (54.4)	2 (2.2)	39 (43.3)
Levofloxacin	≤0.12 to 32	1	≥8	49 (54.4)	4 (4.4)	37 (41.1)
Gentamicin	0.5 to ≥64	≥16	≥16	39 (43.3)	1 (1.1)	50 (55.6)
Amikacin	1 to ≥64	≤2	4	87 (96.7)	0 (0.0)	3 (3.3)
TMP-SMX	≤1 to ≥32	≥16	≥32	10 (11.1)	–	80 (88.9)
Tigecycline	≤0.12 to 2	≤0.5	≤0.5	NA	NA	NA

*TMP-SMX, trimethoprim-sulfamethoxazole; S, susceptible; I, intermediate; R, resistant.*

To examine the inter-test agreement between the methods for determining colistin susceptibility, the essential and categorical agreements and very major error (VME) were evaluated. The essential agreement between the BMD method and VITEK 2 was measured as the difference between MICs ≤ ± 1 log_2_ dilution, using the BMD method as the reference standard. Categorical agreement between the two methods was measured as the percentage of isolates with concordant test results (i.e., intermediate vs. resistant). A VME was defined as inconsistent results, e.g., a colistin-resistant isolate from the BMD method that was considered colistin-intermediate by VITEK 2 (Chen et al., 2014).

### Detection and Sequencing of the *mcr* Genes

Polymerase chain reaction (PCR) amplification of the whole-cell DNA from colistin-resistant isolates (via the BMD method) was performed using previously described primers for *mcr-1*, *mcr-2*, *mcr-3*, *mcr-4*, and *mcr-5* (Rebelo et al., 2018). The PCR products were sequenced.

### Molecular Typing of the *mcr*-Positive *E*. *coli*

The sequence types (STs) of the *mcr*-positive *E*. *coli* isolates were determined by multilocus sequencing typing (MLST) (Wirth et al., 2006). The genetic relationships of the *mcr*-positive *E*. *coli* isolates exhibiting the main STs (≥3 isolates) were further evaluated by arbitrarily primed PCR (AP-PCR) and pulsed-field gel electrophoresis (PFGE), as described previously (Hsueh et al., 2002). Randomly amplified polymorphic DNA (RAPD) patterns of the *mcr*-positive *E*. *coli* isolates were generated by AP-PCR using four primers: M13 (5’-TTATGTAAAACGACGGCCAGT-3’), ERIC1 (5’-GTGAATCCCCAGGAGCTTACAT-3’), ERIC2 (5’-AAGTAAGTGACTGGGGTGAGCG-3’), and 1254 (5’-CCGCAGCCAA-3’) (Operon Technologies, Inc., Alameda, CA, United States). Isolates with RAPD patterns containing one or more discrete bands were considered different.

The DNA extraction and purification for PFGE followed previous descriptions (Tenover et al., 1995; Hsueh et al., 2002). DNA was digested using the *Sma*I restriction enzyme, the restriction fragments were separated using a CHEF-DR III unit (Bio-Rad Laboratories, Hercules, CA, United States), and the pulsotypes were analyzed on the Bio-Rad CHEF-Mapper apparatus (Bio-Rad Laboratories). Cluster analysis was performed in BioNumerics version 5.0 (Applied Maths, Sint-Martens-Latem, Belgium) using the unweighted pair-group method with arithmetic averages. The Dice correlation coefficient was used to analyze similarities between the banding patterns (tolerance = 1%). Isolates with identical PFGE patterns were considered the same strain (same pulsotype) and isolates with PFGE patterns >80% similar were considered closely related. Isolates exhibiting identical ST, RAPD patterns, and pulsotypes, or closely related strains by PFGE, were considered identical strains (clones).

### Identification of the Enterohemolytic *E*. *coli*

Among the 90 isolates, 45 (50.0%) were enterohemolytic *E*. *coli*. The rate of enterohemolytic *E*. *coli* among all of the isolates ranged from 0% in 2013–2014 to 66.7% (*n* = 12) in 2017 (Table 1).

## Results

### Antimicrobial Susceptibilities

The MICs of 13 antimicrobial agents against the 90 porcine *E*. *coli* isolates are shown in Table 2. The susceptibility to ceftazidime and cefepime was 56.7 and 94.4%, respectively, and the susceptibility to ciprofloxacin and levofloxacin were both 54.4%. Only 11.1% of the isolates were susceptible to trimethoprim-sulfamethoxazole. All of the isolates were susceptible to ertapenem, imipenem, and meropenem, and all were inhibited by tigecycline at 2 mg/L. The colistin-resistant isolates and those harboring *mcr-1* displayed similar susceptibilities to ciprofloxacin, levofloxacin, and amikacin (Table 3). The colistin-intermediate isolates had higher susceptibility to gentamicin than the colistin-resistant or *mcr-1* positive isolates (both *p* < 0.0001) (Table 3). The strains did not differ in their resistance to the other antibiotics.

**TABLE 3:** Susceptibility of selected antimicrobial agents against the colistin-intermediate, -resistant, and *mcr*-*1*-carrying *E*. *coli* isolates from sick pigs.

	MIC_90_ (mg/L)			No. (%) of susceptible isolates		
Agent	Colistin- intermediate isolates (*n* = 40)	Colistin- resistant isolates (*n* = 50)	*mcr-*1-carrying isolates (*n* = 39)	Colistin-intermediate isolates (*n* = 40)	Colistin-resistant isolates (*n* = 50)	*mcr*-1-carrying isolate (*n* = 39)
Ciprofloxacin	≥4	16	16	23 (57.5)	26 (52.0)	21 (53.8)
Levofloxacin	≥8	≥8	≥8	23 (57.5)	26 (52.0)	21 (53.8)
Gentamicin	≥16	32	32	24 (60.0)*^,^**	15 (30.0)*	14 (35.9)**
Amikacin	≤2	4	4	39 (97.5)	49 (98.0)	38 (97.4)
TMP-SMX	≥16	≥32	≥32	3 (7.5)	7 (14.0)	4 (10.3)

TMP-SMX, trimethoprim-sulfamethoxazole. *p < 0.05 between colistin-intermediate and colistin-resistant isolates; **p < 0.05 between colistin-intermediate isolates and *mcr-1*-carrying isolates.

**FIGURE 1:**
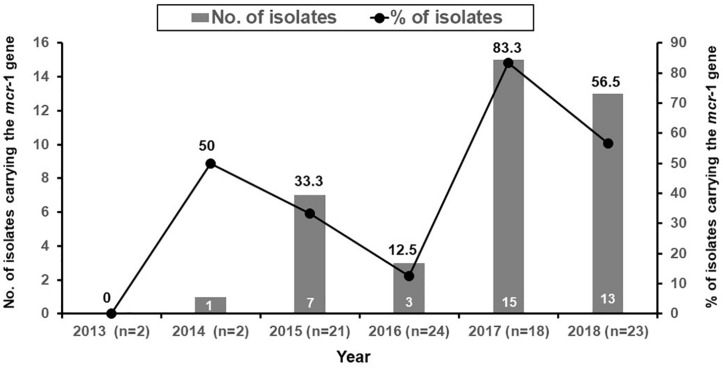
MIC distribution of colistin from the VITEK 2 and reference broth microdilution methods for 90 isolates of *E*. *coli* from sick pigs.

The distribution of colistin MICs determined by the VITEK 2 susceptibility system and the reference BMD method are depicted in Figure 1. The respective rates of colistin-intermediate isolates were 54.1% (*n* = 48) and 44.4% (*n* = 40). The essential agreement of MICs was 97.8%, and the rate of agreement was 91.1% (Table 4). A VME occurred in 16.7% (8/48) of the VITEK 2 susceptibility determinations.

**TABLE 4:** Essential and categorical agreements of the colistin MICs from the VITEK 2 and reference broth microdilution (BMD) methods.

Method	Number (%) of isolates where the VITEK 2-determined MICs differed from the reference BMD MICs according to the number of log_2_ dilutions					No. (%) of isolates	
		
	−2	−1	Same	+1	+2	Essential agreement (%)^*a*^	Category agreement (%)^*b*^
VITEK 2	2 (2.2)	46 (51.1)	28 (31.1)	14 (15.6)	0 (0.0)	88 (97.8)	82 (91.1)

*^*a*^Essential agreement between the BMD broth microdilution method and the VITEK 2 susceptibility testing method was defined as the difference between MICs ≤ ± 1 log_2_ dilution, using the BMD method as the reference standard. ^*b*^Categorical agreement between the two susceptibility testing methods was measured as the percentage of isolates with concordant test results. ^*c*^Seven (8.2%) isolates identified as colistin-resistant with the reference BMD method but were colistin intermediate with the VITEK 2 susceptibility method.*

### Prevalence of the *mcr* Genes Among the Colistin-Resistant *E*. *coli* Isolates

**FIGURE 2:**
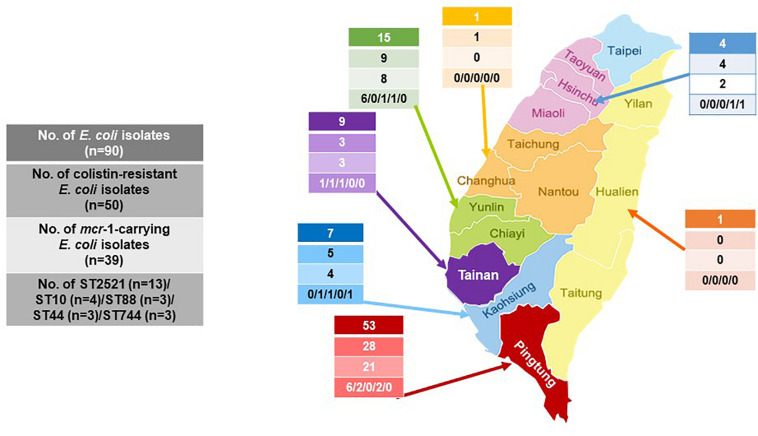
*E*. *coli* isolates harboring the *mcr-1* gene from 2013 to 2018.

**FIGURE 3:**
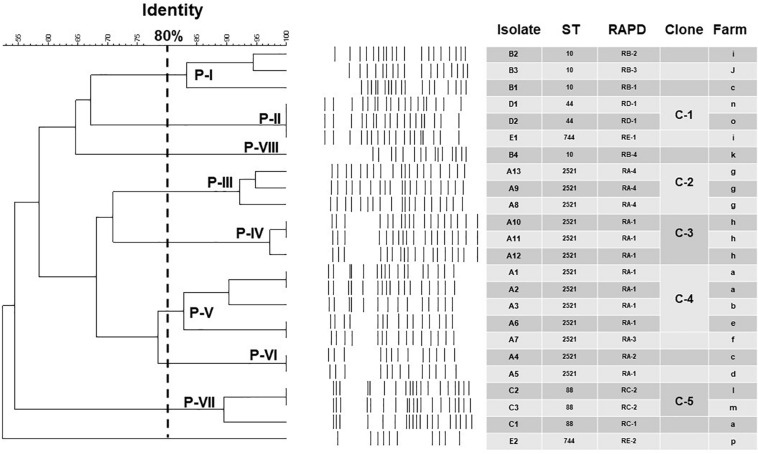
Geographical distribution of the colistin-resistant and *mcr*-*1*-carrying *E*. *coli* and the five sequence types (ST) of *mcr-1 E*. *coli* from 2013 to 2018.

Among the 50 colistin-resistant *E*. *coli* isolates determined by the reference BMD method, 39 (78.0%) were positive for the *mcr-1* gene. The *mcr-2* to *mcr-5* genes were not detected in any of the isolates. *E*. *coli* isolates harboring *mcr-1* comprised 53.3% (24/45) of the enterohemolytic isolates and 33.3% (15/45) of the non-enterohemolytic isolates (*p* = 0.09). The rates of *mcr-1* positive *E*. *coli* isolates rose each year and peaked in 2017 (83.3%) (Figure 2). The respective rates of colistin-resistant and *mcr-1*-positive colistin-resistant *E*. *coli* isolates were 52.8% (28/53) and 75% (21/28) in Pingtung county and 60% (9/15) and 88.9% (8/9) in Yulin county (Figure 3).

### Molecular Typing of the *mcr-1* Isolates

**FIGURE 4:**
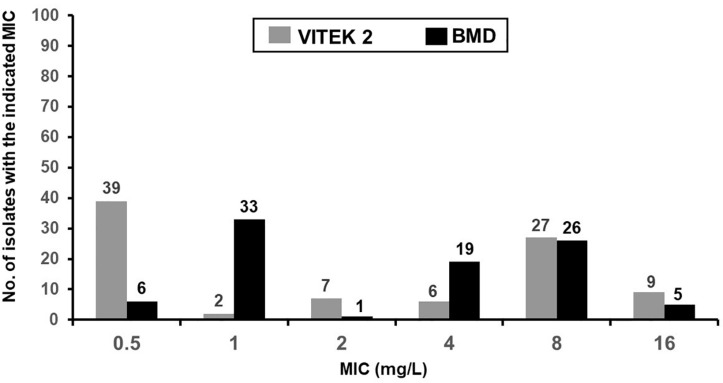
Epidemiological and molecular characteristics of the 24 porcine *E*. *coli* isolates harboring *mcr-1* and displaying five major sequence types (STs) (more than one isolate in each ST). Not included are two porcine *mcr*-*1*-carrying *E*. *coli* isolates (D3 and E3; ST44 and ST744, respectively), different RAPD patterns (RD-2 and RE-3), and different pulsotypes (Clones P-IX and P-X) from the ST44 and ST744 isolates from northern Taiwan (Hsinchu) (see Table 6).

Among the 18 STs identified from the 39 porcine *E*. *coli* isolates harboring *mcr-1*, 13 (33.3%) belonged to ST2521 (all from 2017 and 2018), 4 (10.3%) were ST10, and 3 (7.7%) each were ST88 (all from 2017), ST44, and ST744 (Table 5). AP-PCR and PFGE were performed for all 26 isolates; ST2521 (A1–A13), ST10 (B1–B4), ST88 (C1–C3), ST44 (D1–D3), and ST744 (E1–E3) (Table 6). The isolates were obtained from sick pigs on 18 different farms (identified as farms a to r) (Table 6). A total of 15 RAPD patterns were identified, including ST2521 (RA-1–RA-4), ST10 (RB-1–RB-4), ST88 (RC-1–RC-2), ST44 (RD-1 and RD-2), and ST744 (RE-1–RE-3). PFGE revealed 10 pulsotypes (P-I–P-X) with closely related isolates (Table 6). Five clones (C-1–C-5) were identified from the MLST results, RAPD patterns, and pulsotypes (Figure 4). Three isolates of clone C-2 were from farm g, three isolates of clone C-3 were from farm h, and two isolates of clone C-4 were from farm a. Clones C-1 and C-5 were from different farms.

**TABLE 5:** Distribution of the sequence types (STs) from multilocus sequence typing of the 39 porcine *E*. *coli* isolates carrying the *mcr-1* gene.

ST	No. of porcine *E*. *coli* isolates harboring the *mcr-1* gene						
	2013	2014	2015	2016	2017	2018	Total (% of *mcr*-*1*-carrying isolates)
ST2521	0	0	0	0	6	7	13 (33.3)
ST10	0	0	2	0	2	0	4 (10.3)
ST88	0	0	0	0	3	0	3 (7.7)
ST44	0	0	0	0	0	3	3 (7.7)
ST744	0	0	0	0	2	1	3 (7.7)
ST20	0	0	1	0	0	0	1 (2.6)
ST29	0	0	0	1	0	0	1 (2.6)
ST101	0	0	1	0	0	0	1 (2.6)
ST315	0	0	0	0	0	1	1 (2.6)
ST360	0	0	1	0	0	0	1 (2.6)
ST373	0	0	0	0	1	0	1 (2.6)
ST394	0	1	0	0	0	0	1 (2.6)
ST501	0	0	1	0	0	0	1 (2.6)
ST1771	0	0	0	0	1	0	1 (2.6)
ST4515	0	0	0	1	0	0	1 (2.6)
ST5171	0	0	1	0	0	0	1 (2.6)
ST5229	0	0	0	0	0	1	1 (2.6)
ST8019	0	0	0	1	0	0	1 (2.6)
Total	0	1	7	3	15	13	39 (100)

**TABLE 6:** Characteristics of the 26 porcine *mcr*-*1*-carrying *E*. *coli* isolates exhibiting five major sequence types (STs)*.

No.	Pig farm designation	Isolate designation	Enterohemolytic *E*. *coli*	ST	Source	Date of isolation (year/month/day)	Location	RAPD pattern	Pulsotype^*a*^
1.	a	A1	−	ST2521	Rectal swab	2017/1/16	Yunlin	RA-1	P-V
2.	a	A2	−	ST2521	Rectal swab	2017/1/16	Yunlin	RA-1	P-V
3.	b	A3	+	ST2521	Mesenteric lymph node	2017/5/25	Pingtung	RA-1	P-V
4.	c	A4	+	ST2521	Rectal swab	2017/5/25	Tainan	RA-2	P-VI
5.	d	A5	+	ST2521	Mesenteric lymph node	2017/5/26	Pingtung	RA-1	P-VI
6.	e	A6	+	ST2521	Spleen	2017/7/28	Pingtung	RA-1	P-V
7.	f	A7	+	ST2521	Mesenteric lymph node	2018/1/22	Yunlin	RA-3	P-V
8.	g	A8	+	ST2521	Rectal swab	2018/1/23	Yunlin	RA-4	P-III
9.	g	A9	+	ST2521	Rectal swab	2018/1/23	Yunlin	RA-4	P-III
10.	h	A10	+	ST2521	Mesenteric lymph node	2018/1/30	Pingtung	RA-1	P-IV
11.	h	A11	+	ST2521	Small intestine	2018/1/30	Pingtung	RA-1	P-IV
12.	h	A12	+	ST2521	Small intestine	2018/1/30	Pingtung	RA-1	P-IV
13.	g	A13	+	ST2521	Small intestine	2018/3/5	Yunlin	RA-4	P-III
14.	c	B1	−	ST10	Pericardial cyst	2015/4/24	Tainan	RB-1	P-I
15.	i	B2	+	ST10	Testis	2015/11/21	Kaohsiung	RB-2	P-I
16.	j	B3	+	ST10	Mesenteric lymph node	2017/5/4	Pingtung	RB-3	P-I
17.	k	B4		ST10	Mesenteric lymph node	2017/5/31	Pingtung	RB-4	P-VIII
18.	a	C1	+	ST88	Rectal swab	2017/2/10	Yunlin	RC-1	P-VII
19.	l	C2	+	ST88	Rectal swab	2017/3/29	Tainan	RC-2	P-VII
20.	m	C3	+	ST88	Mesenteric lymph node	2017/8/2	Kaohsiung	RC-2	P-VII
21.	n	D1	−	ST44	Mesenteric lymph node	2018/2/26	Pingtung	RD-1	P-II
22.	o	D2	−	ST44	Mesenteric lymph node	2018/2/26	Pingtung	RD-1	P-II
23.	q	D3	−	ST44	Mesenteric lymph node	2018/6/20	Hsinchu	RD-2	P-IX
24.	i	E1	−	ST744	Spleen	2017/8/11	Kaohsiung	RE-1	P-II
25.	p	E2	−	ST744	Rectal swab	2018/2/27	Yunlin	RE-2	P-VII
26.	r	E3	−	ST744	Colon	2017/5/24	Hsinchu	RE-3	P-X

^*a*^Isolates exhibiting PFGE patterns >80% similar were considered closely related and designated as the same pulsotypes (pulsotypes P-I to P-X). *More than one isolate in each ST is possible.

## Discussion

The prevalence of the *mcr-1* gene in porcine *E*. *coli* isolates from different geographical regions of Taiwan may be unrelated to a specific clonal population. The strains may carry different plasmids encoding the *mcr-1* gene. The MLST of 32 *mcr-1*-positive *E*. *coli* isolates from 18 retail meat samples and 14 human samples in Taiwan (2010–2015) revealed 18 distinct STs, including ST38 (*n* = 8), ST117 (*n* = 5), and two each of ST701, ST744, and ST428. The STs of the remaining isolates were distinct (Kuo et al., 2016). ST744 was also documented in this study, in addition to many other STs. For example, ST2521 was the most common ST, documented since 2017, but it had never been observed in pigs or humans (Kuo et al., 2016; Hadjadj et al., 2017; Kong et al., 2017; Lai et al., 2018; Wang et al., 2020). Our results suggest that further investigation is required to assess the clinical significance of ST2521 among *mcr-1* positive *E*. *coli* isolates. Our findings also indicate that *mcr-1*-positive *E*. *coli* isolates from different geographical regions of Taiwan may have variable STs. The RAPD patterns showed no evidence of clonal dissemination of the *mcr-1-*positive isolates between humans and pigs. In contrast, we identified five clone isolates from the MLST results, RAPD patterns, and pulsotypes. One clone was from sick pigs on different farms and also sick pigs on the same farm. This may indicate intra- and inter-farm spread of porcine *E*. *coli* harboring *mcr-1*. Thus, active and regular screening of *mcr-1*-containing *E*. *coli* isolates from humans and animals is imperative.

A study from China reported 16 different STs among 64 *bla*_*NDM–*__5_- and *mcr-1*-carrying *E*. *coli* isolates from a commercial swine farm (Kong et al., 2017). The five main STs were ST48 (*n* = 12), ST4463 (*n* = 8), ST54 (*n* = 7), ST410 (*n* = 6), and ST165 (*n* = 6). We did not observe any of these STs in our study. Another Chinese study described 40 distinct STs among 58 *mcr*-*1*-positive isolates, indicating considerable diversity among the *mcr-1-*positive isolates from different geographic locations (ST48 and ST10 are widespread) (Yang et al., 2017). In France, six STs (ST4015, ST3997, ST10, ST93, ST48, and ST648) were detected among 25 *mcr-1*-carrying *E*. *coli* isolates from humans, pigs, and chickens (Hadjadj et al., 2017). In the latter study, the ST4015, ST4704, and ST93 porcine *mcr-1*-carrying *E*. *coli* isolates were also different.

Animal-to-human transmission remains a serious concern. A study conducted in Laos reported two cases of colistin-resistant *E*. *coli*; one in a boy with no recent history of antibiotic usage and another in a pig that belonged to the boy’s family. Both isolates belonged to the same novel ST and displayed the same virulence and PFGE patterns (Olaitan et al., 2015). The boy normally fed the pig without protective equipment (e.g., boots). The presence of the same ST4015 *mcr-1*-carrying *E*. *coli* strain in the boy and pig indicates possible horizontal transmission of colistin-resistant *E*. *coli*. Another study described ST648 in two travelers and ST3997 in two villagers, both of which may represent inter-human transmission (Hadjadj et al., 2017). However, the clonality of the 39 *E*. *coli* isolates harboring the *mcr-1* gene was diverse, and 18 different STs were detected (though some STs appeared more than once). Previously, we described six *mcr-1*-carrying *E*. *coli* isolates from patients with bacteremia; two were ST69, but the rest only occurred once (ST1196, ST361, ST1463, and ST1011) (Lai et al., 2018). The human *mcr-1*-positive *E*. *coli* isolates had different pulsotypes, and the STs were different from the porcine *mcr-1*-carrying *E*. *coli* isolates. Although we failed to detect mutual transmission between humans and pigs in Taiwan, we cannot exclude its occurrence. Further surveillance is necessary to identify potential transmissions of the *mcr-1*-carrying *E*. *coli* between animals and humans.

We observed a high VME (16.7%) in the VITEK 2 susceptibility testing of colistin compared to the BMD method. Our findings are contradictory to a previous study in human bacteremic *E*. *coli* isolates (no VME) and indicate that the VITEK 2 method is a low-sensitivity tool for identifying colistin resistance in porcine *E*. *coli* isolates (Lai et al., 2019). Gentamicin is frequently used to treat colibacillosis in pigs, especially in neonatal piglets (via intramuscular or oral administration). Of the 90 *E*. *coli* isolates, the resistance to gentamicin was 55.6% (50/90). High levels of gentamicin resistance were reported in *E*. *coli* isolates from sick pigs in several countries – 32.7% in the United States (Jiang et al., 2019), 46% in Belgium, 45% in Poland, 20% in Spain, and 77% in Korea (Lee et al., 2009; Luppi, 2017). Plasmid-mediated antimicrobial resistance genes are transmissible and cross-resistance between gentamicin and other aminoglycosides such as apramycin (a veterinary drug) has been described (Jensen et al., 2006). However, the prevalence of antimicrobial resistance genes in aminoglycosides requires further investigation.

This study has several limitations. First, a small number of porcine *E*. *coli* isolates were evaluated and all were from sick pigs. This might limit the power of intra- and inter-farm spreading analysis of porcine *E*. *coli* isolates harboring *mcr-1*. The inclusion of more *E*. *coli* isolates from healthy and sick pigs raised on different farms may better describe the prevalence of *mcr-1*-harboring *E*. *coli* in swine and porcine farms. Second, the mechanisms mediating colistin resistance among the *mcr-1*-negative colistin-intermediate *E*. *coli* isolates were not investigated. Third, we investigated *mcr-1* to *mcr-5*, but at least nine *mcr* genes have been reported. The roles of *mcr-6* to *mcr-9* should be investigated.

In conclusion, the occurrence of *mcr-1*-positive *E*. *coli* isolates in sick pigs has continuously increased in Taiwan. Regular screening for the *mcr-1* gene in *E*. *coli* in sick pigs and their environment must be performed to prevent the spread of these resistant organisms.

## Data Availability Statement

The raw data supporting the conclusions of this article will be made available by the authors, without undue reservation.

## Ethics Statement

This study was approved by the Research Ethics Committee of National Taiwan University Hospital (201609066RINB).

## Author Contributions

S-CH, C-CL, and P-RH designed the experiments. S-CH, M-TC, and C-HL executed the lab experiments. S-CH, C-CL, Y-TH, C-HL, and C-NL analyzed the data. S-CH, C-CL, and P-RH prepared the manuscript. S-CH, C-CL, Y-TH, C-HL, M-TC, C-NL, and P-RH read and approved the final version of the manuscript.

## Conflict of Interest

The authors declare that the research was conducted in the absence of any commercial or financial relationships that could be construed as a potential conflict of interest.
